# Primary medullary hemorrhage in a patient with coagulopathy due to alcoholic cirrhosis: A case report

**DOI:** 10.1097/MD.0000000000010292

**Published:** 2018-04-06

**Authors:** Guangxun Shen, Yu Gao, Kwee-Yum Lee, Guangxian Nan

**Affiliations:** a Department of Neurology, China-Japan Union Hospital of Jilin University, Jilin Province, China; b School of Medicine, University of Queensland, Brisbane Australia.

**Keywords:** alcoholic cirrhosis, coagulopathy, etiology, primary medullary hemorrhage

## Abstract

**Rationale:**

Mild-to-moderate alcoholic cirrhosis of the liver is related to spontaneous intracerebral hemorrhage (ICH). In terms of spontaneous brainstem hemorrhage, pontine is considered as the most common site in contrast to medulla oblongata where the hemorrhage is rarely seen. This rare primary medullary hemorrhage has been attributed so far to vascular malformation (VM), anticoagulants, hypertension, hemorrhagic transformation, and other undetermined factors.

**Patient concerns:**

Herein, we describe a 53-year-old patient with 35-year history of alcohol abuse was admitted for acute-onset isolated hemianesthesia on the right side. He was normotensive on admission. A neurological examination revealed isolated hemihypoaesthesia on the right side. He had no history of hypertension, and viral hepatitis, and nil use of anticoagulants.

**Diagnoses:**

Brain computed tomography (CT) image demonstrated hemorrhagic lesion in dorsal and medial medulla oblongata which was ruptured into the fourth ventricle. Brain magnetic resonance imaging (MRI), and magnetic resonance angiography (MRA) demonstrated no evidence of VM. The laboratory tests implied liver dysfunction, thrombocytopenia, and coagulation disorders. Abdominal ultrasound, and CT image showed a small, and nodular liver with splenomegaly, suggestive of moderate alcoholic cirrhosis.

**Interventions:**

Liver protection therapy and the management of coagulation disorders.

**Outcomes:**

After 14 days, he was discharged with mild hemianesthesia but with more improved parameters in laboratory tests. At the 6-month follow-up, brain MRI, MRA, and non-contrast MRI showed no significant findings except for a malacic lesion.

**Lessons:**

We conclude that the patient had alcoholic cirrhosis with coagulopathy, and this may have resulted in primary medullary hemorrhage. This is a first case to report alcoholic cirrhosis as etiology of primary medullary hemorrhage.

## 1 Introduction

There is a modest but independent association between liver disease and the risk of intracerebral hemorrhage (ICH).^[1]^ It is documented that mild-to-moderate alcoholic cirrhosis of the liver is related to spontaneous ICH.^[2]^ ICH can be a sequelae from hematologic complications following cirrhosis, especially from coagulation disorders and thrombocytopenia.^[3]^ Alcoholic cirrhosis has been associated with ICH of cortex, basal ganglia, pons, and cerebellum, but not with medullary hemorrhage, which has never been reported with alcoholic cirrhosis. Herein, we report a patient with primary medullary hemorrhage following coagulopathy due to alcoholic cirrhosis.

## 2 Case report

**Figure 1:**
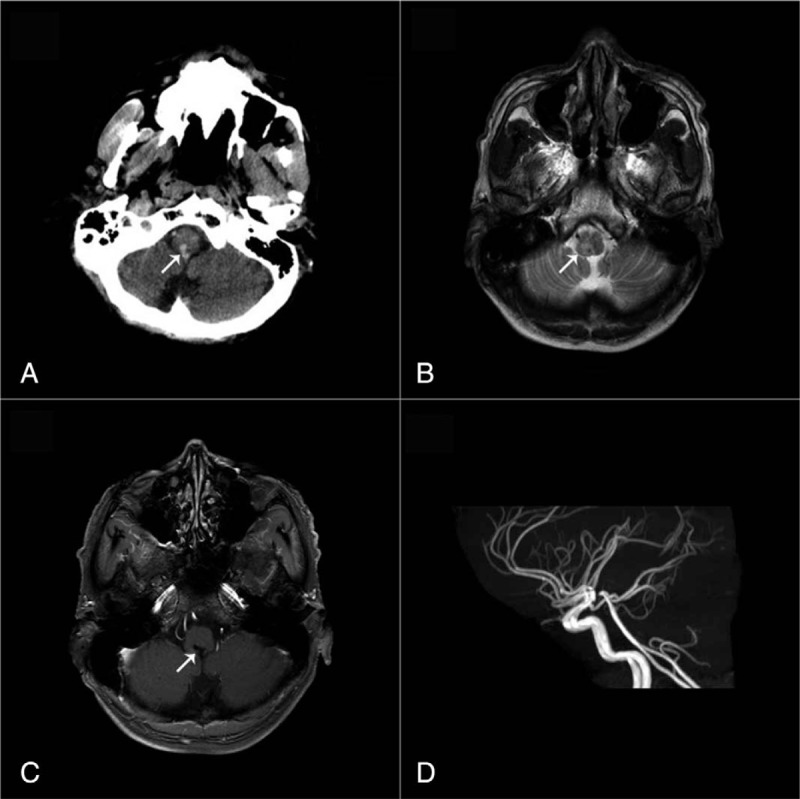
Brain images. Brain CT demonstrated a hemorrhagic lesion in dorsal and medial medulla oblongata with hemorrhage rupturing into fourth ventricle (A; brain MRI with T2 sequences demonstrated hyperintensity in medulla oblongata (B); at 6-month follow-up MRI showed a malacic lesion (C); non-contrast enhanced MRI demonstrated no abnormal areas of enhancement, and negative findings were seen on follow-up MRA (D). CT = computed tomography, MRA = magnetic resonance angiography, MRI = magnetic resonance imaging.

A 53-year-old right-handed man was admitted to our hospital for acute onset of isolated hemianesthesia on the right side. He had no history of hypertension, diabetes, and hepatitis but had 35-year history of daily alcohol consumption of 150 to 250 mL of liquor greater than 50 degrees. On admission, he was normotensive, and, except for hemihypoaesthesia on the right side, a neurological examination revealed no dizziness, nystagmus, dysarthria, facial weakness, tongue deviation, limb weakness, ataxia, Horner syndrome, diplopia, and hearing loss. Deep tendon reflexes were symmetric, and plantar responses were normal bilaterally. Brain computed tomography (CT) demonstrated a hemorrhagic lesion in the dorsal and medial medulla oblongata with hemorrhage rupturing into the fourth ventricle (Fig. 1A). Brain magnetic resonance imaging (MRI) with T2 sequences showed hyperintensity in the medulla oblongata (Fig. 1B), whereas magnetic resonance angiography (MRA) showed no significant findings. Laboratory tests revealed mild coagulation disorders, including mildly elevated prothrombin time (PT) of 13.3 s, thrombin time (TT) of 26.4 seconds, international normalized ratio (INR) of 1.26, and decreased fibrinogen (FIB) at 0.97 g/L. Antigen, and antibody for viral hepatitis, anti-HIV, and syphilis were all negative. Liver function tests showed elevated aspartate aminotransferase (AST) (135 IU/L), alanine aminotransferase (ALT) (104.67IU/L), alkaline phosphatase (ALP) (201.36 IU/L), and gamma glutamyl transferase (GGT) (1547.58IU/L). Count of blood platelet was 57 × 10^9^/L. Abdominal ultrasound, and CT confirmed a small, and nodular liver, and splenomegaly. The score from FibroScan was 18.8 kPa, and that of Child–Pugh was Class A. Oral glutathione was administered with dosage of 0.4 g 3 times daily and hemocoagulase injection with 2 U was started once a day. Fourteen days after the liver protection therapy and the management of coagulation disorders, he was discharged with mild hemianesthesia but with more improved parameters in laboratory tests. At a 6-month follow-up, MRI showed a malacic lesion in the medulla oblongata. Non-contrast enhanced MRI demonstrated no abnormal areas of enhancement, and results showed negative findings on follow-up MRA (Fig. 1C and D).

The patient signed the informed consent form for publication of this case report.

## 3 Discussion

As shown in our case, spontaneous medullary hemorrhage may be followed by alcohol related cirrhosis with thrombocytopenia and coagulopathy. Our patient had no history of hypertension, and never used anticoagulants but reported long-term alcohol abuse. Brain CT revealed isolated medullary hemorrhage. Non-contrast enhanced MRI, and MRA showed no evidence of vascular malformation (VM), and follow-up MRI demonstrated no significant findings except for a malacic lesion. In addition, our case showed that the onset of hemorrhage occurred during the acute mild liver dysfunction with coagulation disorders. With the successful management of liver dysfunction, and coagulation disorders, as well as cessation of alcohol consumption, the medullary lesion shrunk to a malacia.

It has been well documented that alcoholic cirrhosis-related spontaneous ICH is associated with acquired hemostatic deficiency, including thrombocytopenia and coagulopathy.^[4,5]^ The former may arise from hypersplenism, and the latter may be linked to impaired liver function with decreased FIB, and increased fibrinolysis. ICH as a complication of alcoholic liver disease is not uncommon, but hemorrhagic lesions in some specific sites may result from more limited cause, such as VM in midbrain,^[6]^ and medulla oblongata, and cerebral amyloidosis angiopathy (CAA)-related lobar cerebral hemorrhage in elderly patients.

In regard to a spontaneous brainstem hemorrhage, pons is considered to be the most common site, whereas that of medulla oblongata is rarely observed. As such, clinical features, optimal treatment, and prognosis of spontaneous medullary hemorrhage remain unclear,^[7]^ and its etiology has been more limited than that of hemorrhage in other sites. We have searched the PubMed database using ‘medullary hemorrhage (Title/Abstract)’ from the year 1964 up until September 29, 2017. As can be seen in Table 1,^[7–13]^ the search results indicated that medullary hemorrhage has been attributed to VM, hypertension, hemorrhagic transformation, anticoagulants, and undetermined factors. Although it is still challenging to determine causative factors (11 with undetermined etiology of 31 cases), VM is known to be the most common cause (13 of 31 cases). In addition, our search has found that medullary hemorrhage can precede the onset of cavernous angiomas,^[8]^ and hence follow-up brain MRI is necessary. Microaneurysms in the pontine hemorrhage have been demonstrated by autopsy study, but that are absent in the medulla oblongata.^[14]^

**Table 1:**
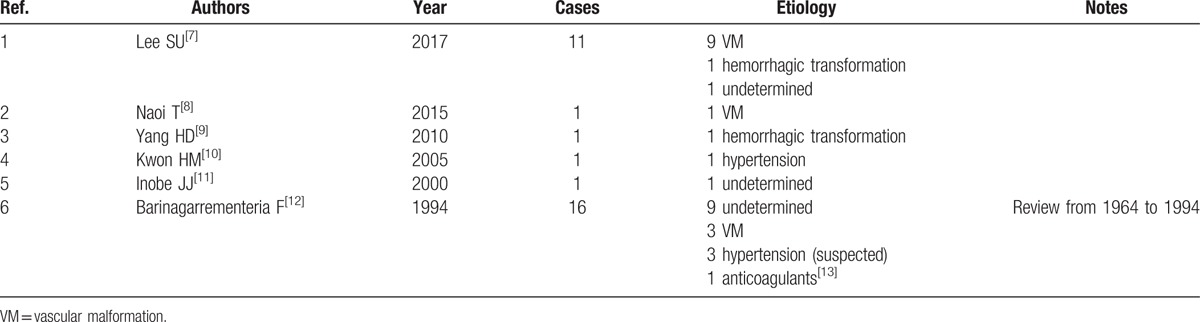
Reported cases of primary medullary hemorrhage.

The fact that isolated medullary hemorrhage is followed by alcoholic cirrhosis with thrombocytopenia, and coagulopathy provides additional cause in primary medullary hemorrhage, which remains to be elucidated.

## 4 Conclusion

We presume that the patient had alcoholic cirrhosis with coagulopathy which may have resulted in primary medullary hemorrhage, which is the first reported case that links alcoholic cirrhosis as etiology of ICH at medulla oblongata.

## Author contributions

**Conceptualization:** Guangxian Nan.

**Supervision:** Guangxian Nan.

**Writing – review & editing:** Guangxun Shen, Yu Gao, Kwee-Yum Lee.
